# Activation of the Parieto-Premotor Network Is Associated with Vivid Motor Imagery—A Parametric fMRI Study

**DOI:** 10.1371/journal.pone.0020368

**Published:** 2011-05-31

**Authors:** Britta Lorey, Sebastian Pilgramm, Matthias Bischoff, Rudolf Stark, Dieter Vaitl, Stefan Kindermann, Jörn Munzert, Karen Zentgraf

**Affiliations:** 1 Institute for Sports Science, Justus Liebig University Giessen, Giessen, Germany; 2 Bender Institute of Neuroimaging, Justus Liebig University Giessen, Giessen, Germany; 3 Institute for Sports Science, University of Berne, Bern, Switzerland; Katholieke Universiteit Leuven, Belgium

## Abstract

The present study examined the neural basis of vivid motor imagery with parametrical functional magnetic resonance imaging. 22 participants performed motor imagery (MI) of six different right-hand movements that differed in terms of pointing accuracy needs and object involvement, i.e., either none, two big or two small squares had to be pointed at in alternation either with or without an object grasped with the fingers. After each imagery trial, they rated the perceived vividness of motor imagery on a 7-point scale. Results showed that increased perceived imagery vividness was parametrically associated with increasing neural activation within the left putamen, the left premotor cortex (PMC), the posterior parietal cortex of the left hemisphere, the left primary motor cortex, the left somatosensory cortex, and the left cerebellum. Within the right hemisphere, activation was found within the right cerebellum, the right putamen, and the right PMC. It is concluded that the perceived vividness of MI is parametrically associated with neural activity within sensorimotor areas. The results corroborate the hypothesis that MI is an outcome of neural computations based on movement representations located within motor areas.

## Introduction

Imagery phenomena have attracted a great deal of attention in the field of cognitive neuroscience during the last decade, and the neural basis of imagery processes has been investigated extensively using behavioral approaches, transcranial magnetic stimulation, and neuroimaging [1]-[4]. All these different approaches have led to the one conclusion that imagery is based on similar brain substrates as the human sensory and motor systems. Hence, motor imagery (MI) is taken to be a simulation that uses motor areas as a substrate [5]. More precisely, this neural network is believed to be organized around the following motor and motor-related regions: the supplementary motor area (SMA), the premotor cortex (PMC), the primary motor cortex (M1), posterior parietal regions such as the inferior (IPL) and the superior parietal lobe (SPL), the basal ganglia (BG), and the cerebellum [3], [6], [7].

Previous work has demonstrated why mentally rehearsing movements has become an important technique in applied sport and exercise psychology for both athletes and patients [8], [9]. In this context, mental practice with MI is used to improve motor task performance and learning [10]. Its benefits, however, often depend on the individual's ability to create vivid motor images. Indeed, there seems to be a relationship between imagery ability and any motor improvement to be seen following MI [11]. Several psychological questionnaires, such as the *Movement Imagery Questionnaire* [12] and the *Vividness of Movement Imagery Questionnaire* [13], have been developed to assess such motor imagery abilities. These subjective reports characterize imagery ability as vividness, that is, the clarity and realism of the respective imagery experience. This process is, for example, associated with the formation and maintenance of the image by working memory. Thus, the vividness of a resulting image reflects the richness of the displayed representation in working memory [14].

Against this background, Guillot et al. [15] have examined how interindividual differences in imagery ability mediate neural activity during MI. Their results demonstrate that good imagers activate motor-related regions such as the posterior parietal and premotor regions to a greater extent than poor imagers. However, up to now, no study using functional magnetic resonance imaging (fMRI) has elucidated what happens in one and the same individual when she or he generates images that differ in their perceived vividness.

Therefore, our goal in the present study was to examine the neural basis of vivid motor images intraindividually with a within-subject correlational approach. We applied a design that asked participants to perform MI of right-hand movements. After each imagery trial, they rated the perceived vividness of every single motor image. Hence, participants rated their imagery performance in terms of its clarity and realism. Finally, brain regions showing increased BOLD signal with increased ratings of perceived imagery vividness were subjected to a parametric analysis. Following the previous literature on MI, we expected the neural activation in motor and motor-related regions, especially in premotor and posterior parietal regions, to relate systematically to perceived vividness of imagery. More specifically, we hypothesized a parametric relationship between the rating of vividness of imagery and the neural activation within motor and motor-related areas.

## Results

### Ratings on perceived vividness of motor imagery

After each imagery trial, participants were asked to evaluate the quality of their imagery performance on a 7-point scale assessing imagery vividness. All participants showed high mean levels of imagery vividness in all imagery conditions: (1) no spatial accuracy, no object, ten repetitions: *M* = 5.30; *SD* = .74; (2) no spatial accuracy, no object, twenty repetitions: *M* = 5.36; *SD* = .86; (3) low spatial accuracy, no object, ten repetitions: *M* = 5.66; *SD* = .70; (4) low spatial accuracy, no object, twenty repetitions: *M* = 5.25; *SD* = .70 ; (5) high spatial accuracy, no object, ten repetitions: *M* = 5.39; *SD* = .62; (6) high spatial accuracy, no object, twenty repetitions: *M* = 5.34; *SD* = .72; (7) no spatial accuracy, object, ten repetitions: *M* = 5.26; *SD* = .82; (8) no spatial accuracy, object, twenty repetitions: *M* = 5.07; *SD* = .82; (9) low spatial accuracy, object, ten repetitions: *M* = 5.46; *SD* = .75; (10) low spatial accuracy, object, twenty repetitions: *M* = 5.23; *SD* = .73; (11) high spatial accuracy, object, ten repetitions: *M* = 5.23; *SD* = .88; and (12) high spatial accuracy, object, twenty repetitions: *M* = 5.17; *SD* = .92. The total rating range comprises values varying between one and seven, with seven demonstrating excellent imagery.

**Figure 1:**
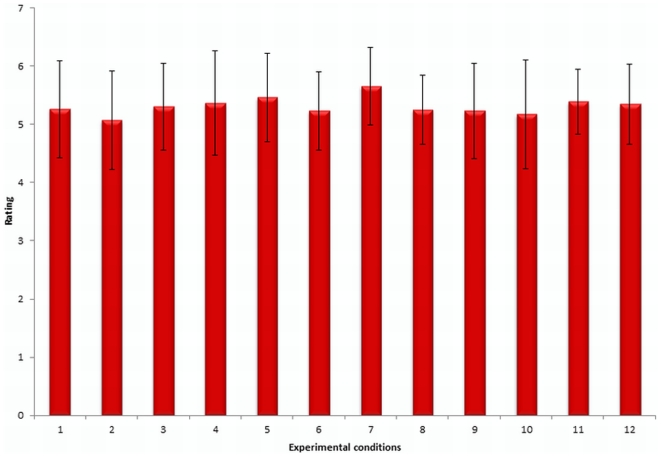
Mean vividness rating score and standard errors depicted for each condition (1: No spatial accuracy, object, 10 repetitions; 2: No spatial accuracy, object, 20 repetitions; 3: No spatial accuracy, no object, 10 repetitions; 4: No spatial accuracy, no object, 20 repetitions; 5: Low spatial accuracy, object, 10 repetitions; 6: Low spatial accuracy, object, 20 repetitions; 7: Low spatial accuracy, no object, 10 repetitions; 8: Low spatial accuracy, no object, 20 repetitions; 9: High spatial accuracy, object, 10 repetitions; 10: High spatial accuracy, object, 20 repetitions; 11: High spatial accuracy, no object, 10 repetitions; 12: High spatial accuracy, no object, 20 repetitions.

A repeated-measures ANOVA revealed no significant difference for vividness of imagery in terms of spatial accuracy, *F*(2, 42) = .858, *p* = .431, η^2^ = .039, object involvement, *F*(1, 21) = 2.62, *p* = .120, η^2^ = .111, or number of repetitions, *F*(1, 21) = 3.255, *p* = .086, η^2^ = .134, and no significant interaction effects(object involvement x spatial accuracy: *F*(2, 42) = .22, *p* = .8, η^2^ = .01; object involvement x number of repetitions: *F*(1, 21) = .151, *p* = .701, η^2^ = .007; spatial accuracy x number of repetitions: *F*(2, 42) = 2.51, *p* = .093, η^2^ = .107; object involvement x spatial accuracy x number of repetitions: *F*(2, 42) = .152, *p* = .230, η^2^ = .068). This indicates that conditions do not differ with respect to the variable of interest, that is, imagery vividness (Fig. 1).

### Neuroimaging Data – Parametric Analysis

**Figure 2:**
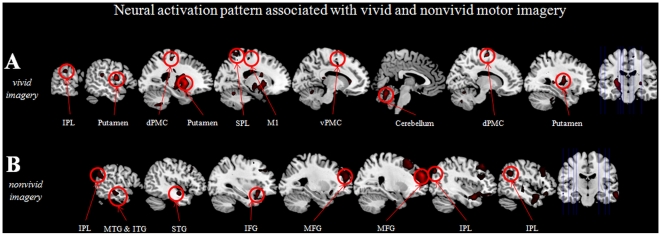
Brain areas showing greater activation as a function of vivid (A) and nonvivid (B) motor imagery based on calculating a parametric modulation.

**Figure 3:**
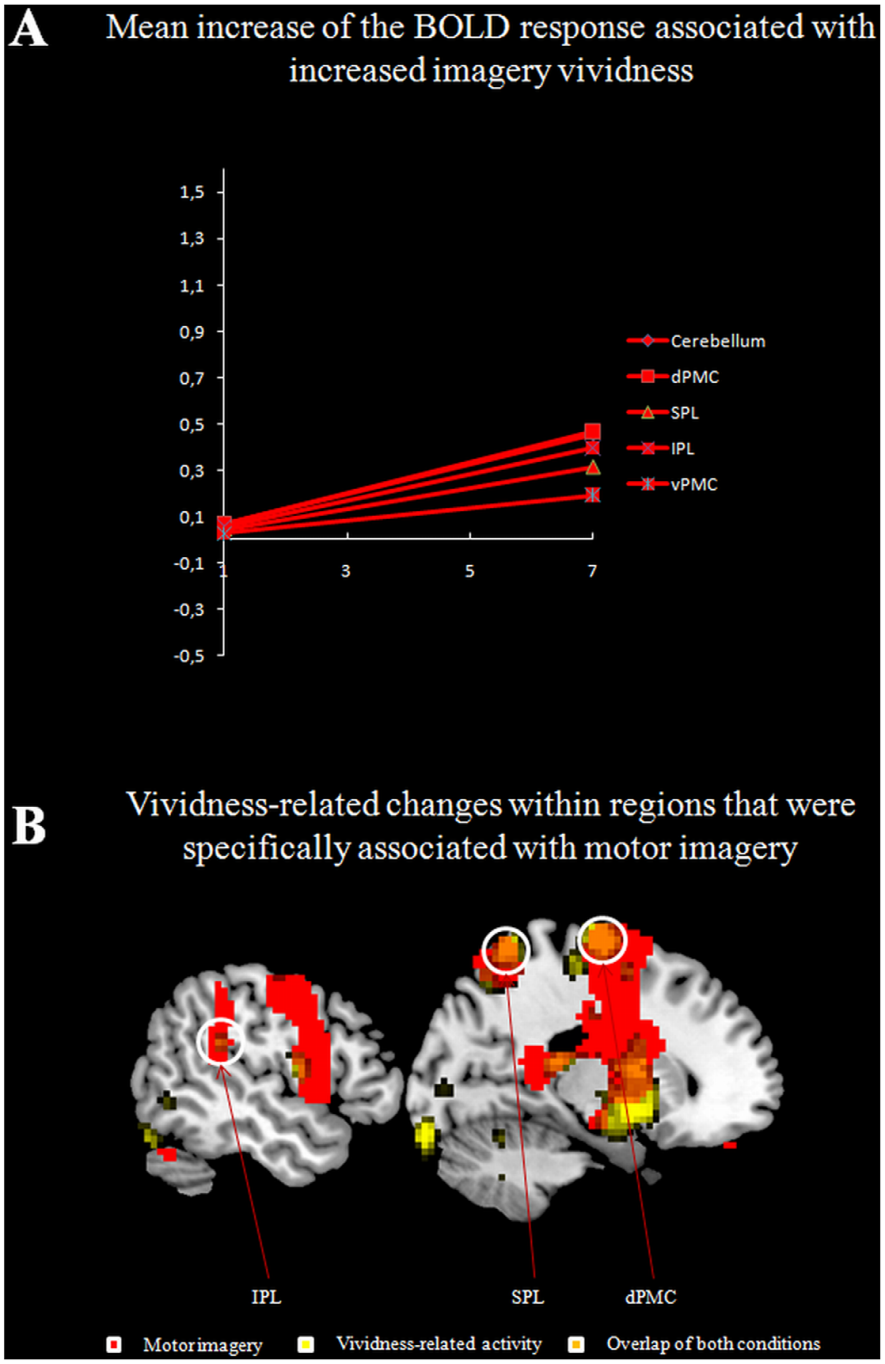
Mean increase of the BOLD response associated with increased imagery vividness (A). Brain areas showing increased activation during motor imagery and as a function of imagery vividness (B).

A parametric analysis was performed to determine which brain sites were modulated by perceived imagery vividness. Results revealed a vividness-dependent increase of activation in a left-hemispheric network capturing the left putamen, the dorsal as well as the ventral part of the left PMC (dPMC and vPMC), the left inferior parietal cortex, the anterior part of the left superior parietal lobe, the left primary motor cortex (M1, Area 4a), the left somatosensory cortex (S1, Area 3b), the left insula, and the left cerebellum (Crus VIIIb). Within the right hemisphere, the activation cluster captured the right cerebellum (Crus VI) and the cerebellar vermis, as well as the right putamen. Another activation site was found in the right dPMC (Fig. 2A). These results are summarized in Table 1.When testing for regions whose activation was associated with vividness-related changes as well as with motor imagery specific effects, we found activation clusters within the superior and inferior parietal, as well as within the dorsal part of PMC (Fig. 3B, Table 2).

**Table 1:** Brain regions showing stronger activation as a function of increased vividness of imagery based on calculating a parametric modulation (*q*<.05, FDR-corrected).

	Left/Right	Coordinates of max. *t* value			*t* value
*Vivid imagery*					
Putamen	L	−24	6	0	6.41
Putamen	R	30	−9	9	5.15
dPMC	R	12	−9	66	6.21
dPMC	L	−24	−24	57	5.14
vPMC	L	−12	0	42	4.29
Cerebellum (VI/Vermis)	R	3	−66	−24	5.42
Cerebellum (VI)	R	27	−60	−24	3.80
IPL	L	−63	−24	30	4.56
IPL	L	−54	−33	21	4.60
SPL	L	−18	−54	69	4.23
M1 (Area 4a)	L	−18	−33	69	4.56
Cerebellum (VIIIb)	L	−21	−60	−48	5.09
Insula	L	−42	−3	3	4.17
S1 (Area 3b)	L	−54	0	15	5.95
V1 (Area 17)	R	36	−54	3	6.07
Nucleus caudatus	R	21	−24	18	5.74
V1 (Area 17)	L	−30	−57	6	5.43
Nucleus caudatus	L	−12	27	6	4.94
Thalamus	L	−18	−33	15	3.82
S1 (Area 3b)	L	−24	−18	30	4.24
Cerebellum, Lobule VI	R	24	−39	−36	4.15

MNI coordinates, FDR-corrected, *q*(FDR) <.05, cluster size >10.

**Table 2:** Brain regions showing stronger activation as a function of increased vividness of imagery that are also specifically activated during motor imagery.

	Left/Right	Coordinates of Max. t Value			t Value
dPMC	L	−18	−12	72	4.84
SPL (7A)	L	−21	−57	69	3.41
IPL (PFcm)	L	−51	−36	24	2.83

MNI coordinates, FWE-corrected, *p*<.05.

Brain areas showing a negative correlation with perceived imagery vividness were the middle frontal gyrus, the pars opercularis of the inferior frontal gyrus, the middle cingulate cortex, the temporal pole, the inferior temporal gyrus, the medial frontal gyrus, the superior orbital gyrus, the hippocampus, and the inferior parietal cortex of the right hemisphere. Other activation sites were detected in the inferior frontal gyrus, the superior temporal gyrus, the middle part of the temporal gyrus, as well as the inferior temporal gyrus and the inferior parietal cortex of the left hemisphere (Fig. 2B). Another activation site was found within the cerebellar vermis.

Thus, it was primarily areas unrelated to the core motor network that depicted a step-wise activation increase associated with decreasing imagery vividness. These results are summarized in Table 3.

**Table 3:** Brain regions showing stronger activation as a function of decreased vividness of imagery based on calculating a parametric modulation (*q*<.05, FDR-corrected).

	Left/Right	Coordinates of max. *t* value			*t* value
*Non-vivid imagery*					
MFG	R	24	54	27	8.40
Inferior temporal gyrus	R	57	−24	−18	7.23
Inferior frontal gyrus	L	−33	18	−15	6.03
Temporal pole	R	42	12	−21	5.57
IPL	L	−60	−54	24	4.72
IPL	R	39	−81	36	5.27
IPL/Angular gyrus	R	63	−54	36	7.33
MFG	L	−27	54	21	4.94
Inferior frontal gyrus (Area 44)	R	48	21	9	3.28
Superior temporal gyrus	L	−45	−6	−12	4.70
Middle temporal gyrus	L	−60	−27	−9	4.36
Inferior temporal gyrus	L	−57	−9	−33	4.07
Middle cingulate cortex	R	6	−45	39	3.90
Hippocampus	R	21	−9	−33	3.79
Superior orbital gyrus	R	21	42	−24	3.63
Cerebellum (Vermis)		0	−42	−33	3.46
V3	L	−39	−93	−15	3.88
Middle cingulate gyrus	L	−15	−48	36	3.58
Inferior temporal gyrus	L	−39	−27	−30	3.65

MNI coordinates, FDR-corrected, *q*(FDR) <.05, cluster size >10.

To ensure that increased neural activation did not result from increased imagery duration, we compared trials with short and long imagery durations. No activation differences were detected. Findings on neural activation differences between imagery conditions with different accuracy demands have been published elsewhere [16].

### Neuroimaging Data – Mean parameter estimates for all conditions

When all parameter estimates of the different imagery conditions were weighted equally, activations were found within the dorsal part of the left PMC. Within the right hemisphere, the activation cluster captured both dorsal and ventral parts of the PMC. Within posterior parietal areas, the activation cluster captured the superior and the inferior part of the parietal cortex (Fig. 3A). Again, we found activation within the cerebellum. This analysis demonstrated that the parametric relationship also persisted after controlling the statistical independence of the different parameter estimates for the different conditions. The results are summarized in Table 4.

**Table 4:** Brain regions identified when weighting all parameter estimates of the different imagery conditions equally.

	Left/Right	Coordinates of Max. t Value			t Value
dPMC	L	−36	−30	63	3.79
dPMC	R	54	−9	54	3.73
vPMC	R	54	−9	48	3.33
SPL	L	−15	−57	69	4.18
IPL	L	−51	−39	24	3.07
Cerebellum	R	36	−57	−12	4.07

MNI coordinates, FWE-corrected, *p*<.05.

For a more detailed insight, Table 5 depicts a breakdown of neural activations within the respective regions of interest (ROIs) for each separate condition. For this analysis, trials with ten and twenty repetitions were pooled. These results demonstrated that none of the conditions make a particularly strong contribution to the given neural activation pattern. The lack of significant activation observed within some conditions might be due to the minor number of volumes measured for each condition.

**Table 5:** Brain regions identified when weighting all parameter estimates of the different imagery conditions equally and depicting significant activations for each condition.

	Cerebellum (L)	Cerebellum (R)	IPL (L)	IPL (R)	SPL (L)	SPL (R)	PMC (L)	PMC (R)
NSA	-	-	-	-	-	-	-	-
LSA	-	-	-	-	-	X	-	-
HSA	-	X	-	-	-	-	X	X
NSA/object	-	-	X	-	X	-	X	-
LSA/object	-	-	-	-	-	-	-	X
HSA/object	-	-	-	-	-	-	-	-

ROI analysis, FWE-corrected, *p*<.05.

## Discussion

The present findings demonstrate a close parametric relationship between activation in human motor areas and the imager's perceived motor imagery vividness. The novelty of the present data comes from its intraindividual, trial-by-trial correlational approach. Our data highlight that subjective data assessed with a psychological evaluation tool (e.g., a rating scale) relate clearly to objective data such as neural activation assessed by fMRI. Moreover, the findings show that imagery vividness is linked parametrically to activation in motor areas, especially within a parieto-premotor network. Thus, our data support the notion that MI is a body-based simulation [17] that relies on the sensorimotor system as an essential substrate. The following sections will discuss these findings in more detail.

### Neural activation within the motor areas and its link to vividness of motor imagery

In the last two decades, a broad body of literature has demonstrated that MI uses the motor system as a neural substrate (for a review, see [7]). The present data are consistent with these well-established findings. As in previous studies, they underpin the importance of parietal, premotor, and cerebellar areas for MI [18], [16], [3]. However, they reveal for the first time a positive correlation between imagery vividness and activation within the motor and motor-related areas in a within-subject design.

A close connection between subjective ratings and subsequent motor performance has been demonstrated in motor learning studies [19], [20]. For example, good imagers, as determined by a questionnaire, require fewer trials to learn new movement patterns. On the neural level, the idea of a close connection between imagery expertise and neural activity within motor-related areas is underpinned by a recent study from Guillot et al. [15]. An extreme group comparison revealed that both poor and good imagers activate similar neural networks that involve motor-related areas. However, in line with our data, good imagers show stronger activation in motor-related areas such as the parietal and premotor cortices. This data nicely shows that vivid or poor imagery relates systematically on an interindividual level to specific activation within the motor system. However, we can now extend these findings by showing that the relationship between vivid MI and activation within parieto-premotor regions is also found in an intraindividual, trial-by-trial correlational approach. Thus, not only does the MI performance of good imagers result in stronger activation of these regions, but also each individual's vivid motor imagery is parametrically associated with higher activation in these areas: the more vivid the motor image, the higher the neural activation within motor and motor-associated areas.

Combining both findings, we suggest that neural activation within the motor network, especially within parieto-premotor areas, varies inter- and intraindividually with perceived motor imagery vividness. Therefore, we believe that psychological assessments and introspection offer a promising and informative method for studying imagery performance because of their connection to neural activity within motor areas.

### A possible relationship between vivid motor images and motor awareness

As stated, motor and motor-related areas such as parietal, premotor, and cerebellar cortices are thought to play a decisive role in the generation of motor images [16], [18]. Recently, a pivotal article by Desmurget and Sirigu [21] has claimed that subjective feelings of conscious motor intention and movement awareness are mediated by a neural network involving posterior parietal as well as premotor areas such as the SMA and the PMC. The present findings show that perceived motor imagery vividness is also linked to the extent of neural activation within these areas. In this regard, we argue that not only motor awareness but also MI are generated within cortical areas that are considered to be responsible primarily for movement planning and motor control. Arguments supporting this view come from computational neurosciences. Here, it has been argued that so-called internal models provide a computational foundation for movement planning and motor control [22]. Within this framework, forward models predict the behavior of a body segment in response to a motor plan. During the last decade, these predictive consequences of forward models have also been thought to mediate motor awareness [21], [23] and cognitive states of movements such as MI [24]. For MI, this hypothesis is based on the assumption that forward models run ‘off-line’ to predict the sensory consequences of the imagined movement.

On a neural level, forward model prediction processes are especially related to activation within posterior parietal, cerebellar, and premotor regions [21], [25]. Interestingly, these regions are known to contain internal representations of both movements and one's own body [26], [27]. In this regard, we suggest that the parametrical association found between the activation within parietal, premotor, and cerebellar regions and the reported motor imagery vividness result from the participant's ability to retrieve an internal movement representation associated with a prediction of the movement's consequences. As stated, activation of the parieto-premotor network is also considered to induce motor awareness. Thus, for both motor awareness and MI, movement execution is not necessary for the vivid feeling of a movement. In fact, this feeling is generated by neural activity within a network associated with the generation of movement intentions and the prediction of each movement's consequences.

One possible flaw in this interpretation would emerge if the discussed effect was actually not associated with imagery vividness but with preparatory motor activity for delivery of the rating. This effect might, e.g., vary with the subjects' certainty about the perceived vividness. However, there are several arguments that make it unlikely that the discussed neural activation increases are associated with preparatory activity in the motor system. First, the rating was delivered with the left hand. The increased neural activation, however, captures foremost left hemispheric motor areas as well as the right hemispheric cerebellum. Yet, activation within these sites is associated with (preparatory) motor activity concerning the right hand. Second, the cross of the rating scale always starts in the middle position. Thus, ‘vivid’ and ‘non-vivid’ ratings are associated with the same number of key-presses. A parametric association between motor preparation of key presses and positive imagery ratings can therefore be ruled out.

### Negative correlations between imagery perceived vividness and neural activation

Although the present study does not inherently focus on brain sites whose activation correlates negatively with perceived imagery vividness, we calculated this contrast in order to control for the specificity of our main hypothesis. The present findings demonstrate a negative parametric relationship between perceived imagery vividness and activation in nonmotor areas located primarily in the frontal and temporal lobe of both hemispheres. First and foremost, these results support our notion that activation in human motor areas is the neural correlate for the imager's perceived motor imagery vividness, and that this interrelation is very specific.

Nonetheless, we also detected activation sites within the inferior parietal cortex of both hemispheres and within the cerebellar vermis. Previous studies have demonstrated their involvement in motor simulation, motor programming, and planning of future acts [3], [16], [28], [29]. The observation in the present study that activation within these areas is also correlated with nonvivid motor images suggests that participants try to start the imageries in the way intended by the given instruction. However, participants did not consider these trials to be successful. This might have been because they experienced a sudden break in the imagery process or realized they had made a mistake. In particular, the activation in frontal areas such as the medial frontal cortex might point to cognitive control or error processing behavior [30].

### Conclusions

We have shown that vivid motor images are associated parametrically with neural activity in motor areas on an intraindividual level. Therefore, we argue that motor images are rooted in the motor system and result from neural computations based on movement representations located within motor and motor-related areas. Regarding potential applications, the present data demonstrate that results stemming from psychological assessments are connected to neural motor processes. This makes it possible to consider their potential as a valid and economic tool for assessing a person's ability to create motor images.

## Materials and Methods

The present study reports data on the neural basis of vivid motor images examined with a within-subject, trial-by-trial correlational approach. Another detailed description of the experimental paradigm as well as a detailed account of the data collected on the neural differences between MI conditions with different movement affordances has been given elsewhere [16].

### Participants

Twenty-three right-handed students (12 female and 11 male, mean age  = 24.49 years, *SD* = 3.01) with normal or corrected-to-normal vision participated in the study. One participant was excluded due to very little variation in perceived imagery vividness.

All participants reported no history of psychiatric or neurological disorders, and no history or current use of any psychoactive medication. The study was approved by the *Ethics Committee of the German Psychological Society* (Deutsche Gesellschaft für Psychologie), and all participants gave their informed written consent in accordance with the Declaration of Helsinki.

### Experimental Procedure

**Figure 4:**
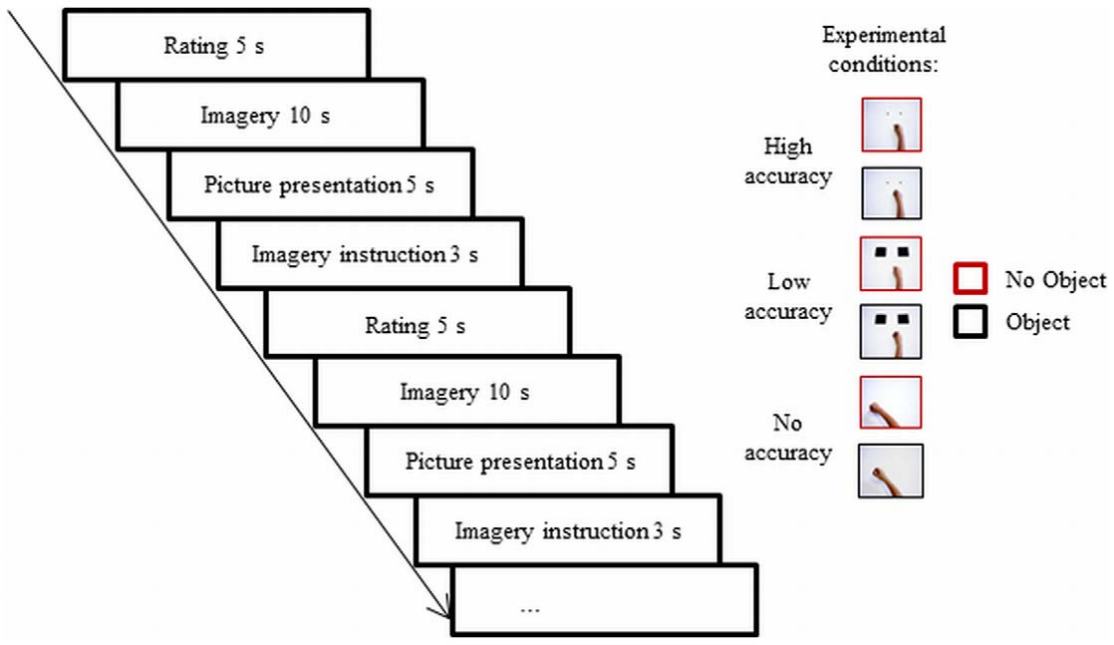
Experimental procedure (left) and experimental conditions (right).

In the fMRI phase, participants were scanned during a rest condition and while performing MI. Six variations of a repetitive aiming task with varying spatial accuracy (no, low, or high) either including an object or not were imagined. The stimulus material consisted of different pictures depicting the setting in which the respective hand movement was to be imagined (see Fig. 4; experimental conditions). These pictures showed: (a) no squares either with an object or without, (b) two big squares either with an object or without, or (c) two little squares either with an object or without. We chose imagery tasks with object-related movements and movements without objects in environments of varying spatial accuracy to ensure that the imagined movements differed in terms of their movement affordances.

The conditions were presented in a pseudo-randomized order counterbalanced across participants. Participants were instructed to imagine placing either their fingers or the object on the right- and left-hand square in alternation. This task resembles a classical Fitts' task paradigm [31]. In trials without squares, they were instructed to imagine a simple repetitive movement in space, again with or without an object in their hands. In one half of the trials, participants were instructed to imagine 10 repetitions of the hand movement; in the other half, 20 repetitions. This allowed us to apply a manipulation check using mental chronometry. Participants always marked the beginning and the end of each MI trial by pressing a key on a button box with their left hand. As shown in prior work, imagery trials with more repetitions and increased accuracy result in increased imagery durations [16]. To ensure that increased imagery duration did not result in increased neural activation, we compared trials of different imagery length.

During MI, participants kept their eyes closed, reopening them only when imagery was over and the button had been pressed. Eye closure and opening were controlled with a video camera. In the rest condition, participants also pressed a button at the beginning and at the end of the rest trial with their left hand. They closed and reopened their eyes in time with the button presses.

In order to assess the perceived vividness of MI, participants were asked how vividly they had experienced their prior imagery (*“How vivid was your imagery performance?”*). Each participant was instructed to rate the imagery as imagery of feeling him- or herself doing the movement. They used their left hand to move the cursor on a 7-point Likert scale with the poles *perfectly clear and vivid* (1) and *only thinking of the movement* (7) to enter their perceived imagery vividness rating immediately after every MI trial.

Each instruction was presented for 3 s. The slide indicating the respective condition was presented for 5 s, and the following MI trial lasted 10 s with no reaction time cutoff. The slide indicating the rating was presented for 5 s [16]. Participants performed 120 imagery trials and 10 rest trials (2 [object: yes vs. no] x 3 [spatial accuracy: no vs. low vs. high] x 20 replications) in one run with a total scanning time of approximately 50 min.

### Training Session

Prior to the fMRI experiment, participants attended a training session in order to familiarize themselves with the different imagery tasks and the experimental setting. The training session had a total duration of 120 min. While performing imagery, surface EMG (Schuhfried, Mödling, Austria) was recorded from two target muscles of the right arm (*M. biceps brachii* and *M. triceps brachii*) to ensure that participants refrained from contracting their arm muscles during imagery [16].

### Image acquisition and analysis

The fMRI data were collected on a 1.5-T whole-body scanner (Siemens Symphony, Erlangen, Germany) with a standard head coil. Structural image acquisition consisted of 160 T1-weighted sagittal images (1-mm slice thickness). For functional imaging, a total of 1,248 volumes were registered using a T2*-weighted gradient echo-planar imaging sequence (EPI) with 25 slices covering the whole brain (slice thickness  = 5 mm; 1 mm gap; TA = 100 ms; TR = 2.5 s; TE = 55 ms, flip angle = 90 degrees; field of view  = 192 mm x 192 mm; matrix size  = 64×64). The orientation of the axial slices was parallel to the AC–PC line. Trial onsets were jittered within a range of ± ½ TR making them consistent with the intertrial interval.

Image preprocessing was carried out using SPM5 (Wellcome Department of Imaging Neuroscience, London, UK). Origin coordinates were adjusted to the anterior commissure. Furthermore, slice-time correction, realignment (sinc interpolation), and unwarping were performed along with normalization to the standard space of the Montreal Neurological Institute brain (MNI brain). Smoothing was executed with an isotropic three-dimensional Gaussian filter with a full-width-at-half-maximum (FWHM) kernel of 9 mm.

The first-level analysis was computed participant-wise using the general linear model. A boxcar function was convoluted with the hemodynamic response function. Both the imagery phases and the rest phases were entered into the model. Boxcar function length covered the respective imagery intervals. Moreover, six movement parameters of the rigid-body transformation of the motion-correction procedure were introduced into the general linear model (GLM) as covariates. The voxel-based time series were filtered by a low-pass (FWHM  = 4 s) and a high-pass filter (time constant  = 256 s).

We examined brain regions showing increased BOLD signal with increased ratings of perceived imagery vividness with a parametric analysis. These parameter values were included as a modulator of the imagery regressor representing the main regressor of the GLM. We investigated the hypothesis by testing the positive correlation between the parameter and brain activation for each participant. To ascertain the specificity of the respective results and to exclude effects that are diametrical to the formulated hypothesis, we also tested the negative correlations between the parameter and brain activation.

In a next step, we entered the resulting parameter estimates into a second-level one-sample *t* test in which the mean estimate across participants at each voxel was tested against zero (random effects model). The statistical threshold was set at a *q* = .05, corrected for multiple comparisons using the false discovery rate (FDR) criterion.

All regions were detected with Automated Anatomical Labeling (AAL) software [32] or, if already mapped cytoarchitectonically, with maps based on cytoarchitectural data [33] with 25% probability.

For a further more specific analysis, we entered each of the six different imagery parameters of the different conditions separately into a first-level model. This analysis allowed us to control the statistical independence of the different parameter estimates for the different conditions. On the second-level, we implemented a flexible factorial design in SPM8. To assess the mean activation parametrically associated with perceived imagery vividness, we conducted a one-sample *t* test while weighting all parameter estimates of the different imagery conditions equally. Furthermore, we conducted one-sample *t* tests for each condition to examine whether all conditions contribute to the given neural activation pattern.

For these analyses, we conducted several small-volume corrections with a priori search volumes. We selected these regions of interests (ROIs) on the basis of the previous analysis and on the basis of findings reported in the literature [15]. The ROIs were posterior parietal areas such as the SPL and the IPL, the dorsal and ventral part of the premotor cortex, as well as parts of the anterior cerebellum. We mapped all ROIs with maps based on cytoarchitectonic data with 50% probability [33]. We created masks for small volume correction using FSL software [34], and tested for significance on the voxel level (*p* = .05, family-wise error (FWE)-corrected).

To assess whether the vividness-related changes occur within the same regions showing specific motor imagery effects, the results of the parametric analysis were masked with the statistical parametric map of the mean imagery activation found in all MI conditions. Within this mask, we performed a region of interest (ROI) analysis. Regions of interest were selected on the basis of the previous results and the main hypothesis of this paper. These were the superior and inferior parietal lobe, the cerebellum, and the dorsal as well as the ventral part of the PMC. Significance was tested on the voxel-level (p = .05, FWE-corrected).

### Behavioral data acquisition and analysis

We gathered subjective ratings of each imagery trial while participants were in the scanner by using a 7-point Likert scale to indicate perceived imagery vividness. We calculated mean rating scores for each experimental condition, and computed a repeated-measures ANOVA to examine the effects of spatial accuracy, object involvement, and number of repetitions on the participants' subjective ratings.

## References

[1] Decety J, Jeannerod M (1996). Mentally simulated movements in virtual reality: Does Fitts law hold in motor imagery?. Behav Brain.

[2] Decety J, Perani D, Jeannerod M, Bettinardi V, Tadary B (1994). Mapping motor representations with positron emission tomography.. Nature.

[3] Lotze M, Montoya P, Erb M, Hülsmann E, Flor H (1999). Activation of cortical and cerebellar motor areas during executed and imagined hand movements: An fMRI study.. J Cogn Neurosci.

[4] Vargas CD, Olivier E, Craighero L, Fadiga L, Duhamel (2004). The influence of hand posture on corticospinal excitability during motor imagery: A transcranial magnetic stimulation study.. Cereb Cortex.

[5] Jeannerod M (2001). Neural simulation of action: A unifying mechanism for motor cognition.. NeuroImage.

[6] Guillot A, Collet C, Nguyen VA, Malouin F, Richards C (2008a). Brain activity during visual versus kinesthetic imagery: An fMRI study.. Hum Brain Mapp.

[7] Munzert J, Lorey B, Zentgraf K (2009). Cognitive motor processes: The role of motor imagery in the study of motor representations.. Brain Res Rev.

[8] Lotze M, Halsband U (2006). Motor imagery.. J Physiol.

[9] Murphy SM (1994). Imagery interventions in sport.. Med Sci Sports Exerc.

[10] Feltz DL, Lander DM (1983). The effects of mental practice on motor skill learning and performance: A meta-analysis.. J Sport Exerc Psychol.

[11] Munroe KJ, Giacobbi J, Peter R, Hall C, Weinberg R (2000). The four ws of imagery use: Where, when, why, and what.. Sport Psychol.

[12] Hall CR, Martin KE (1997). Measuring movement imagery abilities: A revision of the Movement Imagery Questionnaire.. J Mental Imagery.

[13] Roberts R, Callow N, Hardy L, Markland D, Bringer L (2008). Movement imagery ability: Development and assessment of a revised version of the Vividness of Movement Imagery Questionnaire.. J Sport Exerc Psychol.

[14] Baddeley AD, Andrade J (2000). Working memory and the vividness of imagery.. J Exp Psychol Gen.

[15] Guillot A, Collet C, Nguyen VA, Malouin F, Richards C (2008b). Functional neuroanatomical networks associated with expertise in motor imagery.. NeuroImage.

[16] Lorey B, Pilgramm S, Walter B, Stark R, Munzert J (2010). Your mind's hand: Motor imagery of pointing movements with different accuracy.. NeuroImage.

[17] Gallese V (2005). Embodied simulation: From neurons to phenomenal experience.. Phenomenology Cogn Sci.

[18] Gerardin E, Sirigu A, Lehericy S, Poline JB, Gaymard B (2000). Partially overlapping neural networks for real and imagined hand movements.. Cereb Cortex.

[19] Goss S, Hall C, Buckolz E, Fishburne G (1986). Imagery ability and the acquisition and retention of movements.. Mem Cognit.

[20] Rodgers W, Hall CR, Buckolz E (1991). The effects of an imagery training program on imagery ability, imagery use and figure skating performance.. J Appl Sport Psychol.

[21] Desmurget M, Sirigu A (2009). A parietal-premotor network for movement intention and motor awareness.. Trends Cogn Sci.

[22] Miall RC, Wolpert DM (1996). Forward models for physiological motor control.. Neural Netw.

[23] Sirigu A, Daparti E, Ciancia S, Giraux P, Nighoghossian N (2004). Altered awareness of voluntary action after damage to the parietal cortex.. Nat Neurosci.

[24] Blakemore SJ, Sirigu A (2003). Action prediction in the cerebellum and in the parietal lobe.. Exp Brain Res.

[25] Wolpert DM, Kawato M (1998). Multiple paired forward and inverse models for motor control.. Neural Netw.

[26] Aziz-Zadeh L, Damasio A (2008). Embodied semantics for actions: Findings from functional brain imaging.. J Physiol Paris.

[27] Daprati E, Sirigu A, Nico D (2010). Body and movement: Consciousness in the parietal lobe.. Neuropsychologia.

[28] Decety J, Grezes J (2006). The power of simulation: imagining one's own and other's behavior.. Brain Res.

[29] Filimon F, Nelson JD, Hagler DJ, Sereno MI (2007). Human cortical representations for reaching: mirror neurons for execution, observation, and imagery.. NeuroImage.

[30] Ridderinkhof KR, Ullsperger M, Crone EA, Nieuwenhuis S (2004). The role of the medial frontal cortex in cognitive control.. Science.

[31] Fitts PM (1954). The information capacity of the human motor system in controlling the amplitude of movement.. Journal Exp Psychol.

[32] Tzourio-Mazoyer N, Landeau B, Papathanassiou, Crivello F, Etard O, Delcroix N (2002). Automated anatomic labeling of activations in SPM using a macroscopic anatomical parcellation of the MNI MRI single-subject brain.. NeuroImage.

[33] Eickhoff SB, Stephan KE, Mohlberg H, Grefkes C, Fink GR, Amunts K (2005). A new SPM toolbox for combining probabilistic cytoarchitectonic maps and functional imaging data.. NeuroImage.

[34] Smith SM, Jenkinson M, Woolrich MW, Beckmann CF, Behrens TE (2004). Advances in functional and structural MR image analysis and implementation as FSL.. NeuroImage.

